# Supplementary material for: Self-sustained biphasic catalytic particle turbulence
Source: Nat Commun. 2019 Jul 26;10:3333. doi: 10.1038/s41467-019-11221-w (PMC6659658; doi:10.1038/s41467-019-11221-w)
Supplement: Supplementary file 1 — supplementary information [file 41467_2019_11221_MOESM1_ESM.pdf]

Supplementary Material

Self-sustained biphasic catalytic particle turbulence

by Wang, Mathai, Sun.

Supplementary Note 1: Experimental setup

The turbulent flow experiments were performed in the classical (water-based) thermal convection system (Rayleigh-Bénard convection (RBC)). Supplementary Figure. 1 shows a sketch of the experimental setup. The cell consists of a plexiglas side wall with height $H = 400$ mm (aspect ratio 0.5). The working fluid is confined between the copper top plate which is cooled by the water circulating bath (PolyScience PP15R-40) and the copper bottom plate which is heated by the Kapton film heater. Four resistance thermometers (Omega 44131) are embedded into the thin hole drilled evenly distributed along the circumference of the top and bottom plates to probe the local spot temperature of the top and bottom plates, respectively. One small immersion thermistor (the diameter 0.38 mm, the response time 30 milliseconds in liquids, Model GAG22K7MCD419, TE Connectivity Inc.) is inserted into the bulk region which is at the middle height along the vertical direction ($r/R = 0.83, z/H = 0.50$), where (r, z) is the position of the immersion thermistor, and this thermistor is to measure the temperature time-series for comparison between purely plume-driven thermal turbulence and biphasic activity induced active turbulence. To this classical thermal turbulence system, we introduce a minute ($\phi_v \sim 1\%$) volume fraction of a low conductivity liquid (HFE-7000). This kind of liquid is heavier than water and consequently it will spread on the bottom plate at the initial time, and there is a very slight curvature on the bottom which keeps the HFE-7000 around the center. Heat is input through the bottom plate at a constant heat flux while the top plate is kept at a constant temperature.

During the experiment, the working fluid inside the experimental system will induce thermal expansion and biphasic activity, so there will be volume change in the cell. An open reservoir is connected to the cell through a silicone rubber tube which plays a role as expansion vessel (EV).

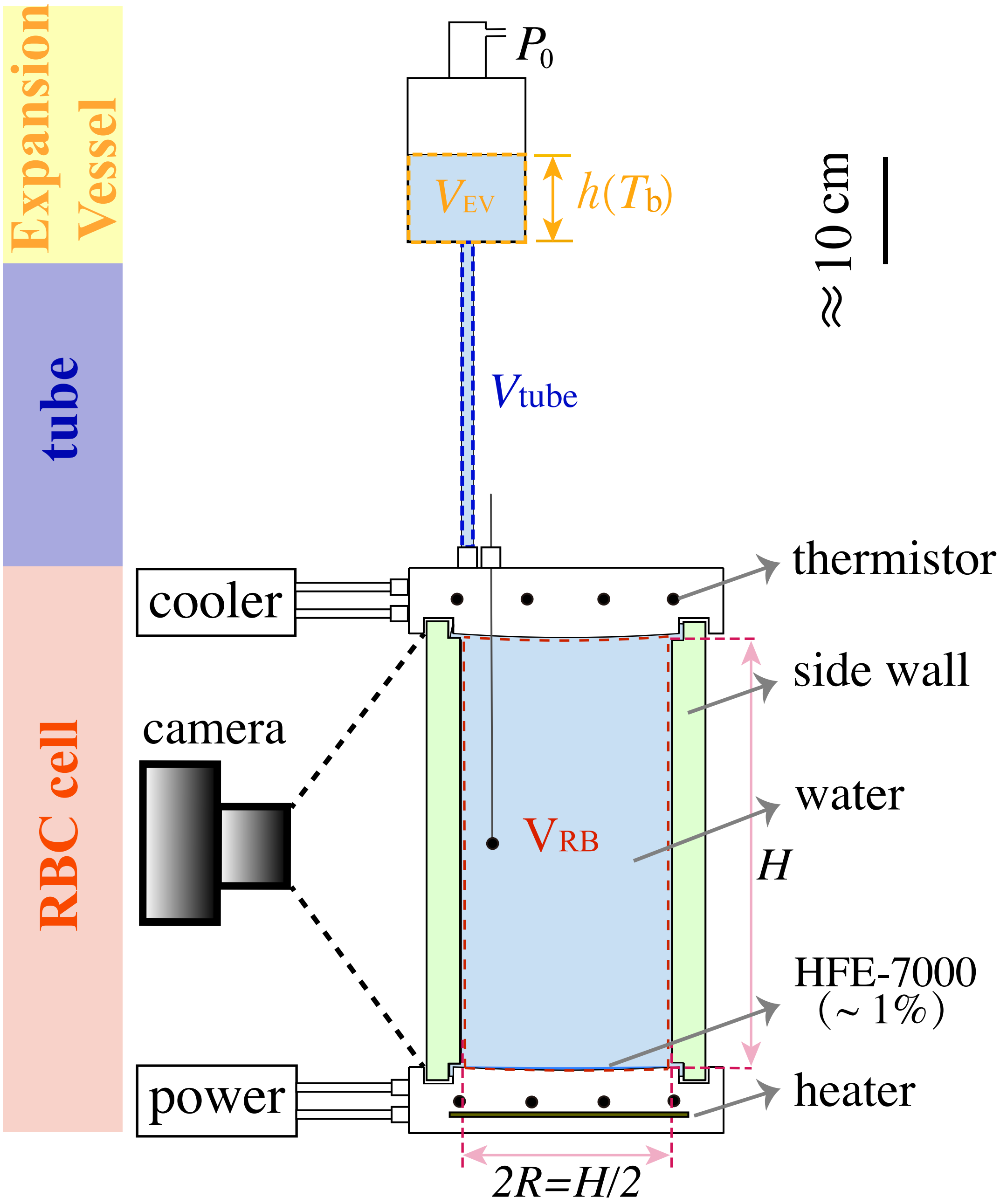

Supplementary Figure 1. Sketch of cylindrical Rayleigh-Bénard convection (RBC) system. There are three sections of the setup involving Rayleigh-Bénard convection (RBC) cell, the expansion vessel (EV) and the tube which connects the RBC cell and EV. The RBC cell is filled with $\sim 1\%$ HFE-7000 liquid (shown as the dark blue layer spreading on the bottom plate) and $\sim 99\%$ water (shown as the light blue occupying large fraction of the RBC cell, whole volume of the tube and part of the EV). The top plate of the RBC cell is cooled by cooler, and the bottom plate is heated. Top plate, bottom plate and PMMA side wall are sealed using silicon O-ring. Four resistance thermometers are inserted into the top and bottom plates, respectively. The EV is open to the atmosphere through the top tube.

The immersion thermistor as shown in Supplementary Figure. 2(a), is comprised of an insulation sheathed wire and the thermal resistance at the tip of the wire coated with very thin glass. The diameter of the thermistor head is about 0.38 mm, and the response time is 30 milliseconds

in liquids which is fast enough for temperature measurements. The thermistor is inserted into the cell through a long thin steel tube and is bend roughly perpendicular to the tube in order to avoid the so-called fin effect. Supplementary Figure. 2(b) is the picture of the immersion thermistor and Supplementary Figure. 2(c) is a sketch for mechanical details.

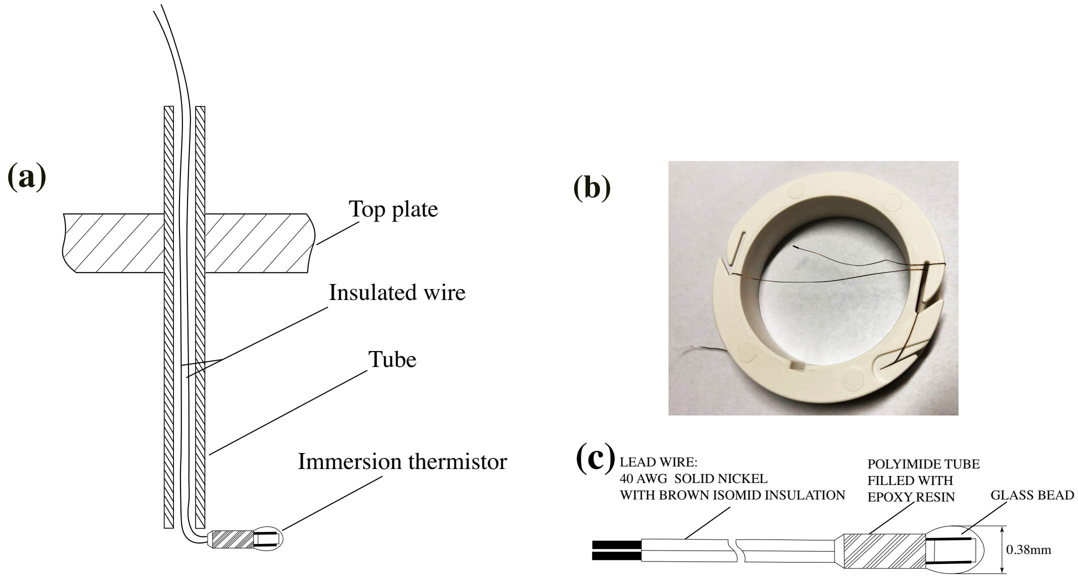

Supplementary Figure 2. (a) Sketch for the way of how the immersion thermistor is inserted into the cell (NTC Temperature Sensor, rapid time response. Model GAG22K7MCD419, TE Connectivity Inc); (b) Photo of the real immersion thermistor; (c) Sketch for the immersion thermistor for mechanical details.

Before the experiment, we fill the RBC cell with $\sim$ 1% HFE-7000 and $\sim$ 99% water, and then add more water until the water level is increasing all through the tube to a certain level inside the EV. The whole system is kept at atmosphere pressure P_0 so that T_{cr} is thought to be constant (T_{cr} is the a critical temperature value above which biphasic "activity" begins to take place). For each bottom plate temperature T_{b}, when the system reaches the stable state the final liquid surface level height $h(T_{\mathrm{b}})$ can be recorded according to which we can calculate the vapor phase of biphasic species volume fraction α. When measuring the heat transfer we use PID (Proportional-Integral-Derivative) controller to control the temperature of different part of the setup (see Supplementary Figure. 3). The setup is placed on an aluminium tub whose temperature is controlled to be the same as T_{b}. The side wall is encircled in an aluminium box that is controlled at mean temperature T_{m} of the cell. The insulation foam fills the gap between the box and the side wall and outside the box wrap several layers of insulation foam. All the insulations and temperature control protocol can prevent the heat exchange between the setup and the environment. After the heat transfer measurement, the insulations are removed and

visualizations are conducted. A high-speed camera is used to record active particle convection regime of the cell and we can use the videos to calculate the large scale circulation (LSC) velocity (V_c).

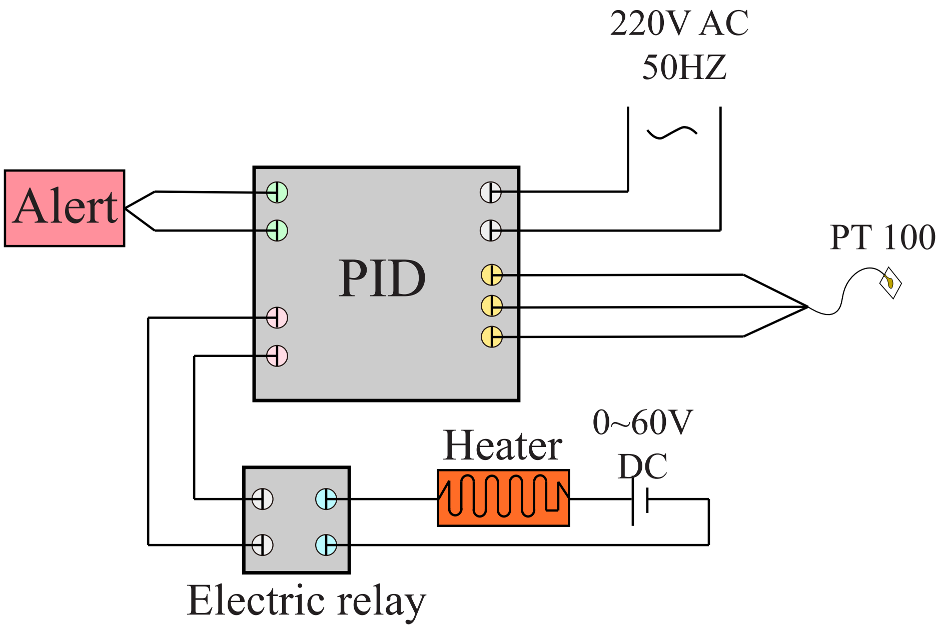

Supplementary Figure 3. Sketch of PID (Proportional-Integral-Derivative) controller. We first need to set the temperature to which we need to reach at the stable state (T_s). PT 100 is put to the spot where we want to control the temperature and is used to measure the temperature T_i which acts as the imported signal into PID system. The PID system will compare the imported temperature with the setted temperature and decide the switch-on and switch-off of the electric relay. The electric relay acts as the switch button that can decide whether the heater will work or not.

Supplementary Note 2: Working fluid

The working fluid is water ($\sim$ 99%). We introduce a minute concentration ($\sim$ 1%) of low conductivity heavy liquid called HFE-7000 liquid (manufactured by 3M^{TM}). HFE-7000 here acts as an "additive catalyst". During the active particle convection process, the biphasic particle is immiscible with water too. Furthermore, the biphasic species (HFE-7000) is non-corrosive, non-flammable and non-ozone depleting (ODP 0), and hence holds promise as a safe and highly effective 'catalyst-like' additive in contemporary clinical, bio-chemical and nuclear engineering settings. The solubility of 60 ppmw HFE-7000 in water can be converted to 43ppmv. The HFE-7000 liquid we add into the system is 1% of the cylindrical cell volume, if we take into account the solubility of HFE-7000 there is 99.57% HFE-7000 by volume which is still remains liquid and contributes to the biphasic catalytic particles and consequently the global heat transfer response.

That is to say only 0.43% of the 1% HFE-7000 liquid (that is $\sim 0.0043\%$ of cell volume) is lost into the water due to miscibility. We expect this to not affect the system much. Water is deionized and ultrapured. Before the experiments, water is boiled twice in order to remove the air dissolved. HFE-7000 is the engineered fluid, 1-methoxyheptafluoropropane, that has a wide use in industrial, pharmaceutical, chemical, electronics applications and so on. Due to low global warming potential (GWP), excellent dielectric properties, low toxicity and so forth, HFE-7000 is of great benefits. It is heavier than water with the density 1.4 kg m^{-3} at 25°C. The critical temperature T_{cr} when the activity begins we observed in the experiment is 41°C which is much lower than that of water, which in industrial means potential for energy savings and more efficient energy usage. All the other physical properties can be find in the product information (3M^{TM} HFE^{TM} 7000 Engineered Fluid (Product Information)) and reference [1] and are given as a function of temperature. During the experiment, all the physical properties related are evaluated at T_{cr} (the experiments were conducted at fixed pressure). Before the experiments, it is boiled to degas.

Supplementary Note 3: Control and response parameters

The control parameters for the self-sustained active particle turbulence are Jakob number Ja, Rayleigh number Ra, Prandtl number Pr and the aspect ratio which describes the geometry of the experimental setup AR. The response parameters is Nusselt number Nu, .

Jakob number Ja_{b} (Ja_{t}) is the ratio of available thermal energy to the energy necessary for generating liquid phase vaporization (latent heat). The subscript 'b' is bottom which means the temperature difference used in calculation of Jakob number is from the bottom plate temperature, 't' top which means the temperature difference used in calculation of Jakob number is from the top plate temperature

$$\text{Ja}_{\text{b}} = \frac{\rho_{\text{Fl}} C_{\text{pF}} (T_{\text{b}} - T_{\text{cr}})}{\rho_{\text{Fv}} L}, \tag{1}$$

$$\text{Ja}_{\text{t}} = \frac{\rho_{\text{Fl}} C_{\text{pF}} (T_{\text{t}} - T_{\text{cr}})}{\rho_{\text{Fv}} L}, \tag{2}$$

where ρ_{Fl} denotes the density of HFE-7000 liquid, C_{pF} the heat capacity per unit mass of HFE-

7000 liquid, $T_{\rm b}$ the temperature of the bottom plate, $T_{\rm cr}$ the critical temperature value above which the biphasic activity begins, $\rho_{\rm Fv}$ the density of HFE-7000 vapor, L, $T_{\rm t}$ the temperature of the top plate.

Rayleigh number Ra denotes the dimensionless temperature difference

$$\mathrm{Ra} = \frac{g\gamma \Delta T H^3}{\nu\kappa}, \tag{3}$$

where g the gravitational acceleration, γ the isobaric thermal expansion coefficient,ν the kinematic viscosity and κ the thermal diffusivity. All of these material and physical properties are calculated for water, which is the working fluid of the system.

Prandtl number Pr is the ratio of viscous diffusion rate to the thermal diffusion rate which is given as

$$\mathrm{Pr} = \frac{\nu}{\kappa}. \tag{4}$$

For a cylindrical experimental cell, the geometry parameter is defined as

$$AR = \frac{d}{H}, \tag{5}$$

where d is the cell diameter, H thickness of the working fluid layer (also height of the experimental cell which is a constant value for a given cell).

In the Rayleigh-Bénard convection cell, the HFE-7000 liquid is of minute volume fraction ($\sim 1\%$), while the two kind of fluid are still stratified with the upper layer water, the down layer HFE-7000. In the classical Rayleigh-Bénard convection, the non-dimensional heat flux which is the ratio of convective to conductive heat transfer across (normal to) the boundary acting as the response parameter of the system Nusselt number Nu is defined as

$$\mathrm{Nu} = \frac{Q}{\lambda_{\rm f} \cdot \Delta T / H} = \frac{Q}{k_{\rm f} \cdot \Delta T}, \tag{6}$$

with Q the measured heat input through the bottom plate into the system per unit time, $k_{\rm f}$ the heat transfer coefficient of pure conduction whose inverse is the overall thermal resistance of pure conduction, and $k_{\rm f} = (H/\lambda_{\rm f})^{-1}$ ($\lambda_{\rm f}$ the thermal conductivity of the working fluid) and $\Delta T = T_{\rm b} - T_{\rm t}$ the temperature difference between the bottom and top plate.

However, in active particle system, the stratified fluids will induce change in the heat transfer coefficient of pure conduction. Here, we treat the pure conduction case as the cascades of the two layer of fluids, i.e. HFE-7000 and water. So the overall thermal resistance is composed of two parts, the resistance inside the HFE-7000 liquid layer and inside the water layer. Therefore, we correct the Nusselt number as

$$\mathrm{Nu} = \frac{Q}{k_{\mathrm{eff}} \cdot \Delta T}, \tag{7}$$

here k_{eff} is the effective heat transfer coefficient of pure conduction, defined as $k_{\mathrm{eff}} = (H/\lambda_{\mathrm{eff}})^{-1} = (h_{\mathrm{w}}/\lambda_{\mathrm{w}} + h_{\mathrm{N}}/\lambda_{\mathrm{N}})^{-1}$ (λ_{eff} is the effective thermal conductivity of the two layer of fluids, h_{w} and h_{N} the height of water layer and HFE-7000 layer respectively in the quiescent regime, λ_{w} and λ_{N} the thermal conductivity of water and HFE-7000 liquid respectively).

For all the heat flux measurements in the experiments, we use the Nusselt number with effective heat transfer coefficient.

Supplementary Note 4: Experiments

The turbulent flow experiments were performed in the cylindrical Rayleigh-Bénard convection (RBC) system shown in Supplementary Figure. 1. We did overall heat flux measurement experiments and dye visualization experiments.

The experiment we showed in the paper is at the condition that $\Delta T \approx 30$ K; we choose this setting is because that Ra= $g\beta\Delta T H^3/\nu\alpha$, where the fluid physical properties are function of mean temperature T_{m}, though T_{m} is changing yet the combination of the physical properties only has slight change, so ΔT controls the main trend of Ra, if ΔT is fixed we can confine Ra almost unchanged (in the experiment, for $\Delta T \approx 30$ K, we have Ra $\sim 4.5 \times 10^{10}$). We know in the classical one-phase RBC Ra is the control parameter which reflects the natural convection driving force intensity; here in the two-phase experiments we confine the natural convection driving force and investigate the effect of two-phase dynamics; therefore we do the experiment on the condition that Ra almost unchanged. And all the heat flux measurements are performed under statistically stationary state after running the experiment for about 10 hours. Below we show an additional figure as an example, Supplementary Figure. 4, showing the sustainability

of the process. The standard deviation of the Nu time series is 0.028 which is accurate enough for the heat transfer measurement, the long time behaviour of the particle (bubble generated) system is stable and self-sustainable.

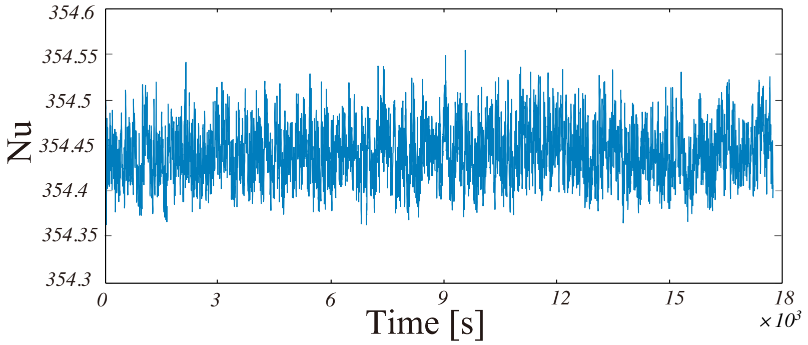

Supplementary Figure 4. Nusselt number time serious of statistically steady state of $T_b - T_{cr} = 5.27K$.

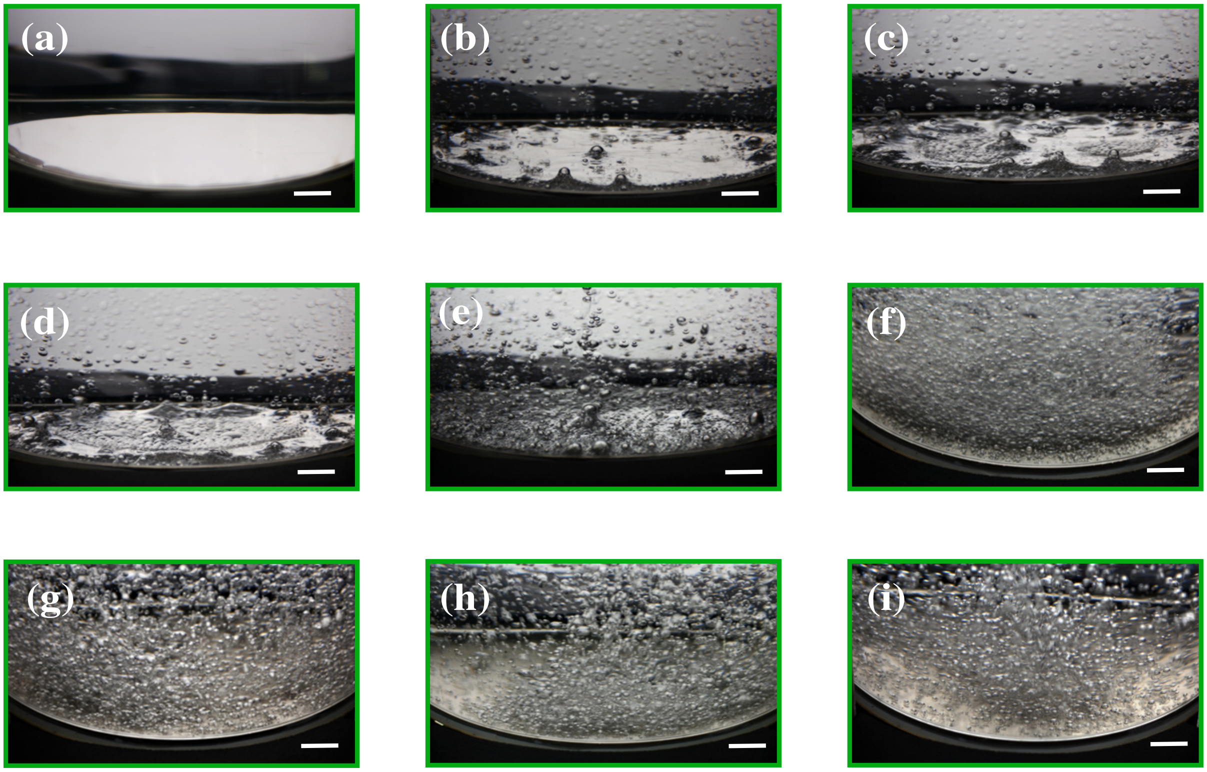

Supplementary Figure 5. Zoom-in time sequence snapshots of bottom plate. The top plate is kept at temperature $T_t \approx 21°C$ by water circulating bath, and the heat input power is set to reach the final bottom plate temperature $T_b \approx 51°C$. (a)-(i) are the snapshots during the changing process. Scale bar: 20 mm.

As the bottom plate temperature T_b is increased from below to above the critical temperature T_{cr}, we can observe the evolution of the HFE 7000 on the bottom plate. As shown in Supplementary Figure. 5, from (a) to (i) , the top plate temperature is kept constant ($T_t \approx 21°C$), at the

meantime, the bottom plate experiences increasing temperature from $T_\mathrm{b} \approx 35$°C to $T_\mathrm{b} \approx 51$°C. (a) is in quiescent regime, so in the cell there is only liquid phase with water ($\sim$ 99% by volume) that occupies most of the volume and HFE-7000 ($\sim$ 1% by volume) spreading on the bottom plate surface, and the heat transfer is induced by classical thermal turbulence. (b)-(f) are in partially active regime (0 K $< T_\mathrm{b} - T_\mathrm{cr} <$5 K), in this regime there is still a fraction of HFE-7000 that is in liquid phase that spreads on the bottom plate, a small fraction of HFE-7000 nucleates, as the increasing level of bottom plate temperature, more and more HFE-7000 begins to take part in the biphasic activity. (g)-(i) are in fully active regime where all HFE-7000 takes part in the activity, but this does not mean all HFE-7000 liquid has become vapor, rather all HFE-7000 has join the bulk region and contributes to the heat transfer enhancement. In fact there is only small fraction of HFE-7000 liquid becomes vapor, the real evaporation fraction is small because the risers are biphasic and the real mass is mostly liquid (once the vapor bubbles detach from the bottom, they dynamically condense while rising towards the top plate). This is further verified by the volume conservation technique. We use the expansion vessel based calculation to get α with measurement error of about 3%, which is precise enough to determine the bubble volume fraction. Within the boiling regime of the experiments, only 6% to 46% of the initial HFE-7000 liquid evaporates. In Supplementary Figure 5(i) we can observe the “sweeping mode”, which is further clarified in the supplementary movies.

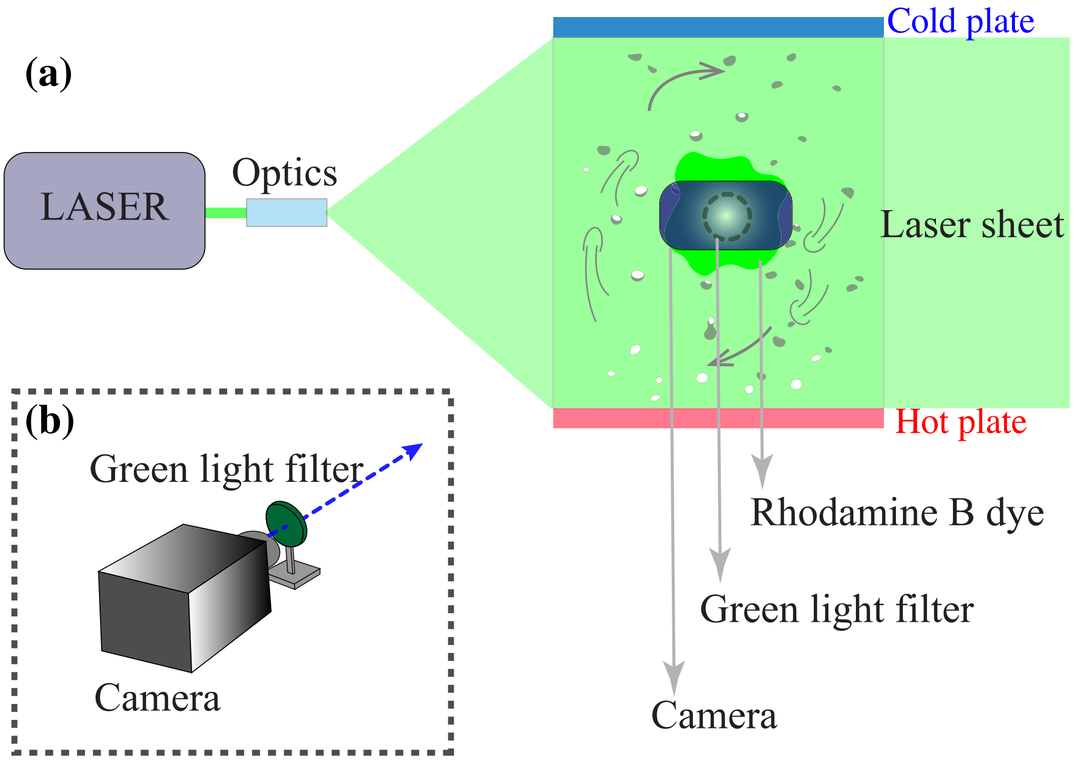

Supplementary Figure 6. Experimental arrangement for dye visualization. (a) Experimental setting sketch of dye visualization. A passive scalar (Rhodamine B dye) was released into the system. The dye is illuminated using a pulse laser (Vlite-200 532nm Solid State Laser System), and a camera is attached with a high-pass (green) light filter to mask stray reflections from the particles. The optics part is to change a laser beam into a layer of laser sheet. False coloring was adopted based on the intensity of the dye in Suppl. Video 9 & 10. (b) Details of the setting of the camera and the green light filter.

After the heat transfer measurements, in order to visualize the mixing activity by active biphasic particles, we release a passive scalar (Rhodamine B dye [2]) into the system. The dye is illuminated using a pulse laser (Vlite-200 532nm Solid State Laser System). We use a camera (HiSense Zyla camera from DANTEC DYNAMICS) to record the images of the mixing processes and the camera is attached with a high-pass (green) filter to mask stray reflections from the particles. Supplementary Figure. 6 shows the experimental arrangement of the dye visualization experiment. A laser generator shoots laser beam pulses which travel through the optics to form laser sheet. The laser sheet shines at the RBC cell. Rhodamine dye is injected into the experiment cell and it will shine green light under the effect of the laser sheet. A high-pass (green) filter is placed in front of the camera so that we can only see the dye mixing dynamics in RBC cell. The laser beam pulses and the camera can be synchronized and thus the camera can freeze the image in the pulse duration of the laser pulses (7 ns). For comparison we conduct two experiments, one is the classical thermal turbulence system dye mixing visualization and

the other is the active biphasic turbulence dye mixing visualization. The experimental settings for the two experiments are the identical: the injected dye is of the same concentration and dose and is injected at the same spot of the cell with the same injecting speed; the bottom plate temperature is 43°C for both cases. From the two experiments we can compare the mixing intensity, and clearly see the difference qualitatively between the one-component one-phase and two-component two-phase RB system. Supplementary Figure. 7 (a,b) show image sequences where the background turbulence advects and mixes the fluorescent dye in traditional thermal convection and biphasic particle-laden turbulence, respectively. In the classical system, the dye mixes along the path of the LSC (see Supplementary Figure. 7(a) and Suppl. Video 3). In contrast, the biphasic system displays fast and chaotic mixing (Supplementary Figure. 7(b)) over a wide range of length scales (see also Suppl. Video 4).

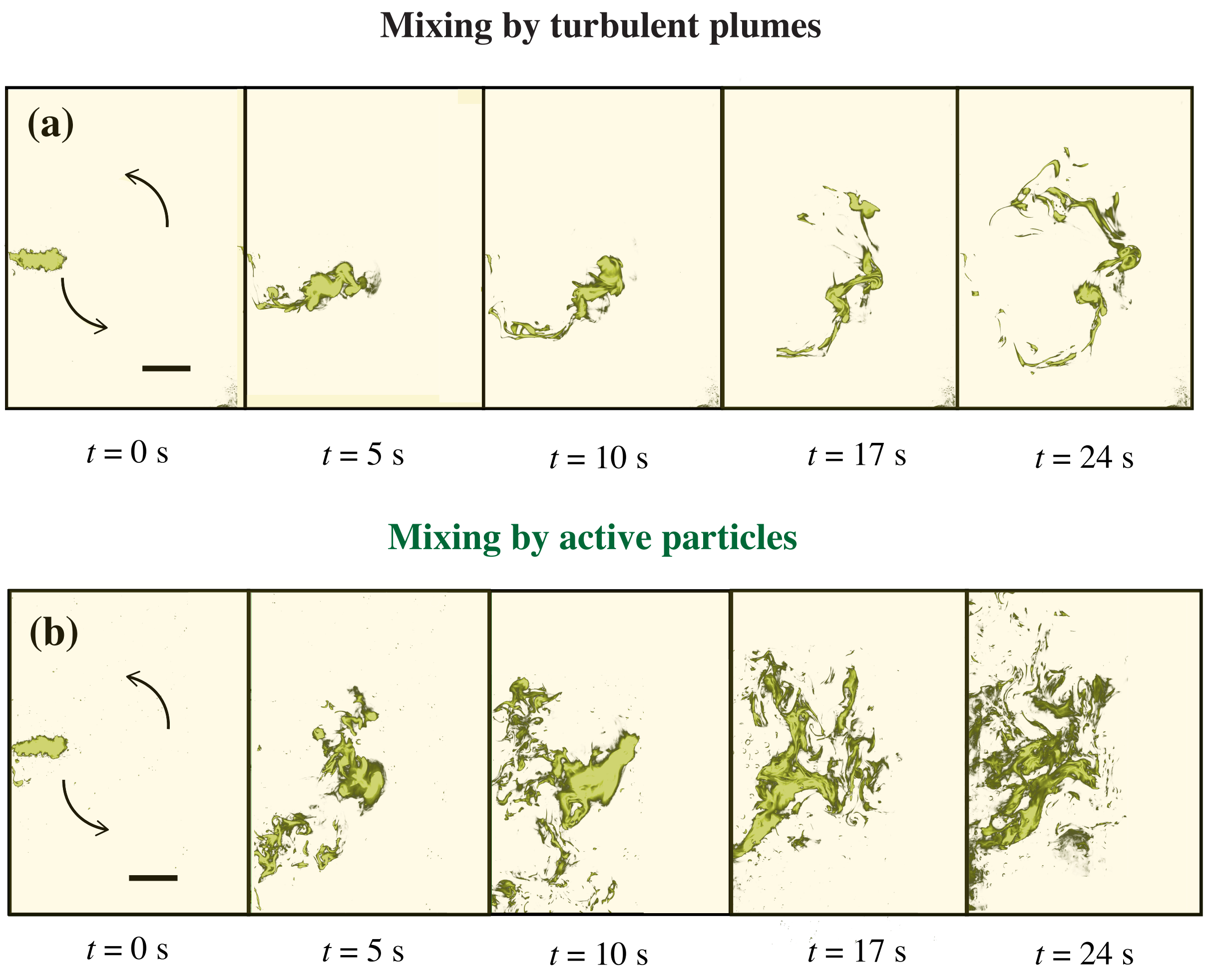

Supplementary Figure 7. Snapshots of mixing of a patch of fluorescent dye (passive scalar) released in (a) classical thermal turbulence system and (b) active biphasic turbulence, at time instants (left to right) 0, 5, 10, 17, and 24 s after injection. In classical thermal turbulence (a), the spreading occurs primarily along path of the large scale circulation (LSC), while in the active biphasic case (b), the mixing is fast and occurs across a wide range of scales (see Suppl. Video 9 & 10 for the comparison). False coloring was adopted based on the intensity of the dye

Next we quantify the wide range of mixing length scales. We notice that the typical structure size in single phase convection is limited to a large scale roll and the plumes. However, in biphasic case, we observe small scale mixed parcels of dye and also large scale, e.g. the large scale circulation, the plumes, biphasic particles and their induced fluid agitation. These different structures all contribute to the global heat transfer. We measured the mean value for all kinds of sizes of dye patches between 15-30 seconds after dye injection, and compared this to single phase case. Supplementary Figure. 8 shows the histogram and the cumulative histogram of all kinds of scales of mixing structure areas A normalized by the vertical cross section area of the cell $A_0 = d \times H$, where d and H are the diameter and the height of the experimental cell respectively. Compared with single phase regime, the biphasic regime displays richer mixing structures, which

indicates fast and chaotic mixing over a wide range of length scales.

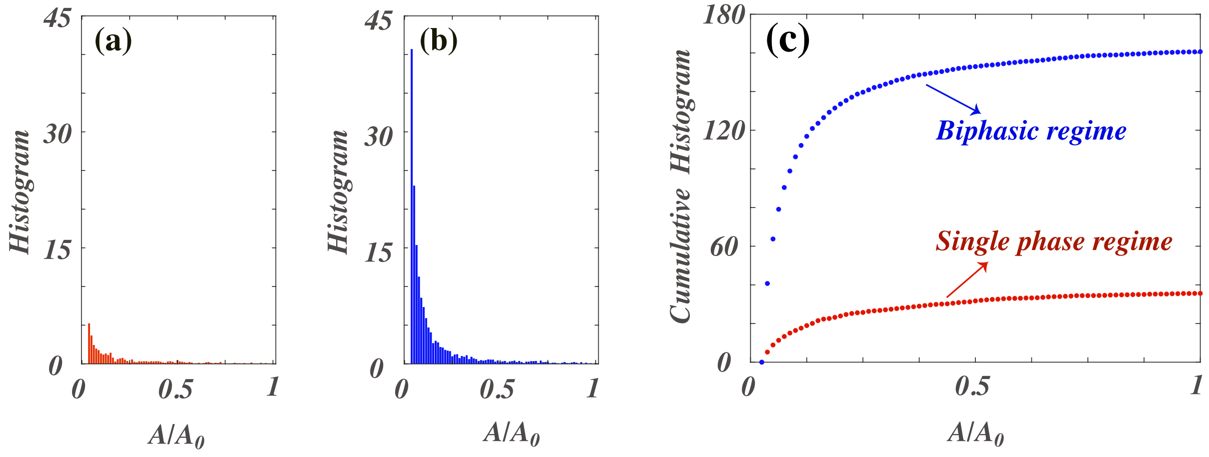

Supplementary Figure 8. (a) Histogram for normalized single phase regime mixing structure area; (b) histogram for normalized biphasic regime mixing structure area; (c) cumulative histogram of the normalized structure areas for single phase regime and biphasic regime.

Next we use the measured the temperature time-series to compare between purely plume-driven thermal turbulence and biphasic activity induced active turbulence. We perform a Fourier analysis of those temperature fluctuations as shown in Supplementary Figure. 9. We can see from the Supplementary Figure. 9(a) that in the single phase regime the temperature fluctuation frequency mostly distributes in the range between 0 $\sim$ 0.3 Hz but centralized around 0.016 Hz and the fluctuation amplitude is high; Supplementary Figure. 9(b) is in the partially active regime, the frequency distributes mostly between the range 0 $\sim$ 0.25 Hz and is centralized around 0.06 Hz and the amplitude is the smallest among the three regimes; Supplementary Supplementary Figure. 9(c) is in the fully active regime, the frequency distributes mostly between the range 0 $\sim$ 0.4 Hz and is centralized around 0.004$\sim$0.036 Hz and around 0.125 Hz, while the amplitude is in between the single phase and the partially active regimes.

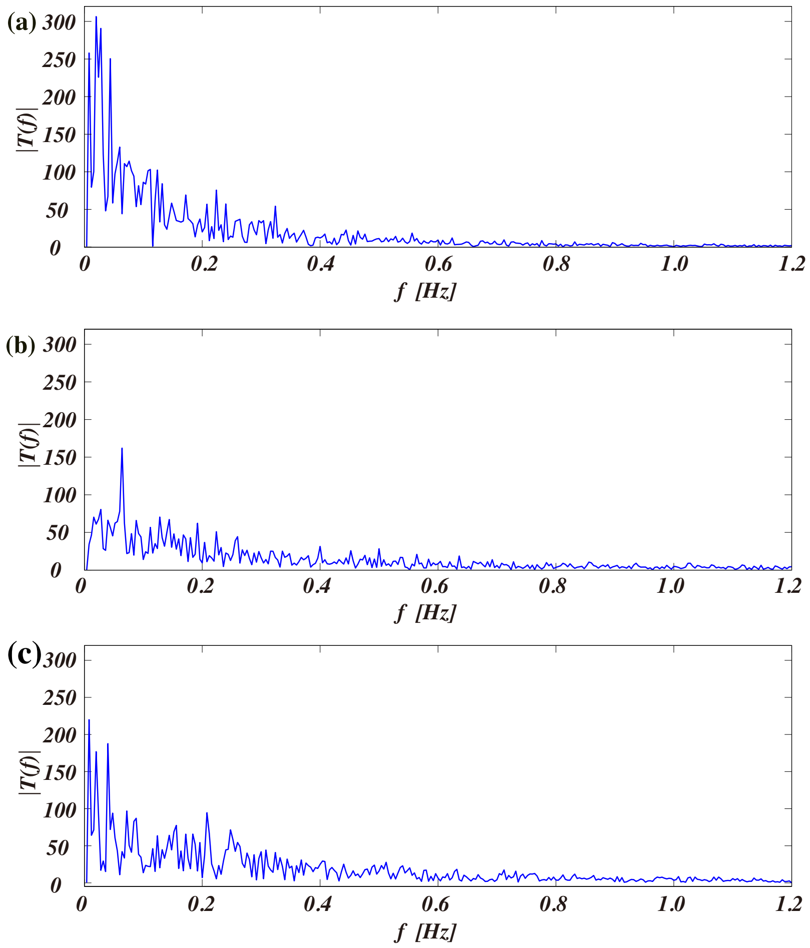

Supplementary Figure 9. Fourier analysis of temperature fluctuations.

Supplementary Note 5: Calculating vapor phase of biphasic species volume fraction α

For each bottom plate temperature T_{b}, when the system reaches the finally dynamically stable state, we have a water surface level height $h(T_{\mathrm{b}})$ inside the expansion vessel (EV), according to which we can calculate vapor phase of biphasic species volume fraction α. Here we define the system as the dashed line (yellow, blue and red) enclosed region in Supplementary Fig. 1. Supplementary Table 1 shows the notations and the descriptions of all the parameters related.

Supplementary Table 1. Parameter notations in calculation of α

Notation	Description
R	radius of RBC cell, constant
H	thickness of the working fluid layer (also height of RBC cell), constant
r	radius of EV, constant
$h(t)$	height of water surface in EV, measured at dynamically stable state for each T_{b}
V_{RB}	volume of RB cell, constant
$V_{\mathrm{RB\cdot w}}(t)$	volume occupied by water in RB cell at time t during the experiment
$V_{\mathrm{RB\cdot Nl}}(t)$	volume occupied by HFE-7000 liquid in RB cell at time t during the experiment
$V_{\mathrm{RB\cdot Nv}(t)}$	volume occupied by HFE-7000 vapor in RB cell at time t during the experiment
$\rho_{\mathrm{w}}(T)$	density of water, $T = T_{\mathrm{m}}$ inside RBC cell, $T = T_0$ inside tube and EV
ρ_{Nl}	density of HFE-7000 liquid evaluated at boiling temperature T_{cr}, constant
ρ_{Nv}	density of HFE-7000 vapor evaluated at boiling temperature T_{cr}, constant
m_0	total mass inside the system (RBC cell+ tube + EV), constant
$m_{0\cdot\mathrm{N}}$	the total mass of the HFE-7000 liquid and vapor during the experiments, constant
$m_{\mathrm{RB}}(t)$	total mass inside RBC cell at time t
$m_{\mathrm{RB\cdot w}}(t)$	mass of water inside RBC cell at time t
$m_{\mathrm{RB\cdot Nl}(t)}$	mass of HFE-7000 liquid inside RBC cell at time t
$m_{\mathrm{RB\cdot Nv}}(t)$	mass of HFE-7000 vapor inside RBC cell at time t
m_{tube}	mass of water inside tube, constant
$m_{\mathrm{EV}}(t)$	mass of water inside EV at time t
α	$V_{\mathrm{RB\cdot Nv}}/V_{\mathrm{RB}}$, vapor phase of biphasic species volume fraction

Before the experiment, the system stays at room temperature T_0 which is controlled by the conditioner and doesn't change throughout the experiment. The water in the tube and the EV is always at equilibrium state with the surrounding environment, which means the water in the tube and the EV has constant temperature T_0. When the bottom plate temperature T_{b} is less than T_{cr}, the water and HFE-7000 inside the RBC cell, the water inside the tube and EV go

through isobaric thermal expansion which causes water level rise in EV. When $T_{\mathrm{b}} > T_{\mathrm{cr}}$, the HFE-7000 liquid boils to form vapor bubbles, and the vapor bubbles rise through the bulk region all the way towards the top plate whose temperature is always below T_{cr}, so the bubbles will experience condensation and isothermal expansion, which means the temperature of HFE-7000 liquid and vapor bubble is fixed at boiling temperature T_{cr}.

We use the fundamental law of physics, namely, conservation of mass, to solve the problem. During the experiments, water and HFE-7000 in the cell experience isobaric thermal expansion and phase change, both of which will induce redistribution of the mass in the system.
As the bottom plate temperature increases, there are two regimes which are quiescent regime and active particle convection regime corresponding to non-boiling and boiling process respectively.

At the quiescent regime, the working liquids go through isobaric thermal expansion, the mass of the system (RBC cell + tube + EV) is conserved, which yields

$$\begin{aligned} m_0 &= m_{\mathrm{RB}}(t) + m_{\mathrm{tube}} + m_{\mathrm{EV}}(t) \\ &= m_{\mathrm{RB\cdot w}}(t) + m_{\mathrm{RB\cdot Nl}}(t) + m_{\mathrm{tube}} + m_{\mathrm{EV}}(t) \\ &= \rho_{\mathrm{w}}(T_{\mathrm{m}}) \cdot V_{\mathrm{RB\cdot w}}(t) + \rho_{\mathrm{Nl}} \cdot V_{\mathrm{RB\cdot Nl}}(t) + m_{\mathrm{tube}} + \rho_{\mathrm{w}}(T_0) \cdot \pi r^2 \cdot h(t). \end{aligned} \tag{8}$$

The mass of HFE-7000 liquid and vapor (in fact, at quiescent regime all HFE-7000 is in the form of liquid phase, so $V_{\mathrm{RB\cdot Nv}} = 0$ which means $\alpha = 0$ ($T_{\mathrm{b}} < T_{\mathrm{cr}}$)) in RBC cell is conserved, which gives

$$\begin{aligned} m_{0\cdot \mathrm{N}} &= m_{\mathrm{RB\cdot Nl}}(t) \\ &= \rho_{\mathrm{Nl}} \cdot V_{\mathrm{RB\cdot Nl}}(t). \end{aligned} \tag{9}$$

The volume of RBC cell is conserved, that is

$$V_{\mathrm{RB}} = V_{\mathrm{RB\cdot w}}(t) + V_{\mathrm{RB\cdot Nl}}(t). \tag{10}$$

At the active particle convection regime, there is phase change as well as isobaric thermal expansion. So HFE-7000 is in the form of liquid phase and vapor phase. the mass of the system

(RBC cell + tube + EV) is conserved, which yields

$$
\begin{aligned}
m_0 &= m_{\mathrm{RB}}(t) + m_{\mathrm{tube}} + m_{\mathrm{EV}}(t) \\
&= m_{\mathrm{RB\cdot w}}(t) + m_{\mathrm{RB\cdot Nl}}(t) + m_{\mathrm{RB\cdot\ Nv}}(t) + m_{\mathrm{tube}} + m_{\mathrm{EV}}(t) \\
&= \rho_{\mathrm{w}}(T_{\mathrm{m}}) \cdot V_{\mathrm{RB\cdot w}}(t) + \rho_{\mathrm{Nl}} \cdot V_{\mathrm{RB\cdot Nl}}(t) + \rho_{\mathrm{Nv}} \cdot V_{\mathrm{Nv}}(t) + m_{\mathrm{tube}} + \rho_{\mathrm{w}}(T_0) \cdot \pi r^2 \cdot h(t).
\end{aligned} \tag{11}
$$

In RBC cell, there is a mixture of HFE-7000 liquid and vapor whose total mass is conserved, which gives

$$
\begin{aligned}
m_{0\cdot\mathrm{N}} &= m_{\mathrm{RB\cdot Nl}}(t) + m_{\mathrm{RB\cdot Nv}}(t) \\
&= \rho_{\mathrm{Nl}} \cdot V_{\mathrm{RB\cdot Nl}}(t) + \rho_{\mathrm{Nv}} \cdot V_{\mathrm{RB\cdot Nv}}(t).
\end{aligned} \tag{12}
$$

The volume of RBC cell is conserved, that is

$$
V_{\mathrm{RB}} = V_{\mathrm{RB\cdot w}}(t) + V_{\mathrm{RB\cdot Nl}}(t) + V_{\mathrm{RB\cdot Nv}}(t). \tag{13}
$$

All equations combined, we find the general form of the vapor phase of biphasic species volume fraction α as

$$
\begin{aligned}
\alpha &= \frac{V_{\mathrm{RB\cdot Nv}}}{V_{\mathrm{RB0}}} \\
&= \frac{\frac{m_0}{V_{\mathrm{RB}}} + \rho_{\mathrm{w}}(T_{\mathrm{m}}) \cdot \left[\frac{m_{0\cdot\mathrm{N}}}{V_{\mathrm{RB}}\cdot\rho_{\mathrm{Nl}}} - 1\right] - \frac{m_{0\cdot\mathrm{N}}}{V_{\mathrm{RB}}} - \frac{m_{\mathrm{tube}} + \rho_{\mathrm{w}}(T_0)\cdot\pi r^2\cdot h(t)}{V_{\mathrm{RB}}}}{\rho_{\mathrm{w}}(T_{\mathrm{m}}) \cdot (\frac{\rho_{\mathrm{Nv}}}{\rho_{\mathrm{Nl}}} - 1)}.
\end{aligned} \tag{14}
$$

$h(t)$ is the only time dependent value and we can measure for each T_{b} when the system arrives at the finally dynamically stable state, and at the stable state $h(t) = h(T_{\mathrm{b}})$ according to which we can calculate α in biphasic regime. Supplementary Figure. 10 show an example of the calculation on the condition that ΔT is fixed throughout the experiment.

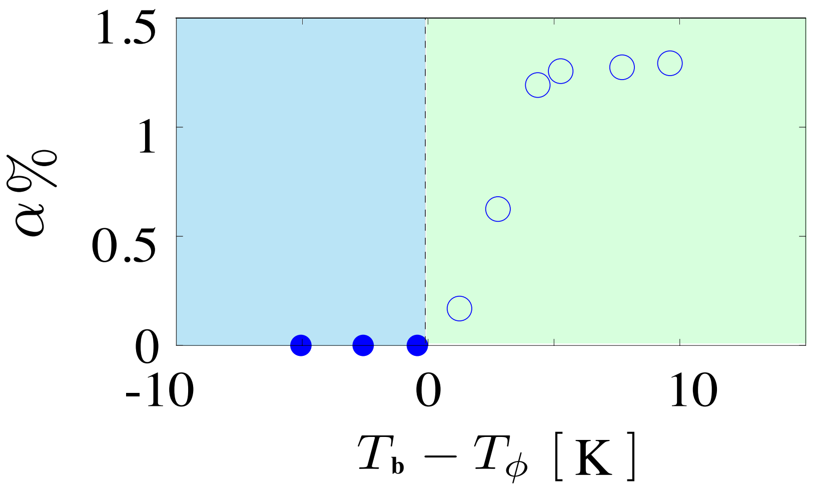

Supplementary Figure 10. Vapor phase of biphasic species volume fraction α as function of $T_\mathrm{b} - T_\mathrm{cr}$. The experiments were conducted at fixed bottom and top plate temperature difference $\Delta T = T_\mathrm{b} - T_\mathrm{t} = constant$ in order to maintain Rayleigh number almost unchanged.The blue shaded area is the quiescent regime and the green shaded area is the active biphasic regime.

The expansion vessel is with scales from which we can read the water surface height h. The scale on the expansion vessel has the minimum scale value 1mm, which corresponds to 0.007% of α. For each case, after the system reaches the finally statistically stable state and we then conduct about four-hour heat flux measurements. During the heat flux measurements, we monitor h from the expansion vessel scales every other 30 minutes and we in total obtain eight readings of h. On top of this, the minimum and maximum variation in α during the four hours measurement was 1.3% variance of α. The value h we use to calculate α is the mean value of the eight readings.

Supplementary Note 6: Calculation of biphasic kinematics contribution

In the active particle convection regime, remarkable heat transfer enhancement are observed, and there are many different mechanisms contributing to such huge enhancement, e.g. biphasic kinematics provides extra heat transfer mechanism in multiphase system, here we can quantify the contribution. In the finally dynamically stable state, we know in the RBC cell the vapor phase of biphasic species volume fraction is α, assuming that all the heat absorbed by the bubbles is for HFE-7000 vapor generation while the temperature of vapor remains same as T_cr. So the vapor bubble mass flux in the vertical direction from bottom to top plate can be estimated, we

have

$$\dot{m}_{\mathrm{v}} = \alpha \cdot \pi d^2/4 \cdot \rho_{\mathrm{v}} \cdot V_{\mathrm{c}}, \tag{15}$$

where $\dot{m}_{\mathrm{v}}$ is the vapor bubble mass flux (unit: kg s^{-1}) across the horizontal cross section of the RBC cell, $\pi d^2/4$ measures the cross section area of the system, here the parameter d is the diameter of the cell, V_{c} is the characteristic collective bubble velocity scale (unit: m^{-1}).

The heat carried by the vapor bubble mass flux can be obtained

$$Q_{\mathrm{l}} = L \cdot \dot{m}_{\mathrm{v}}, \tag{16}$$

with Q_{l} the heat flux induced by biphasic kinematics mechanism (unit: W), L the latent heat of HFE-7000 vapor (unit:J kg^{-1}).

So the contribution of biphasic kinematics mechanism to the non-dimensional heat flux enhancement Nu_l is as follows

$$Nu_{\mathrm{l}} = \frac{Q_{\mathrm{l}}}{k_{\mathrm{eff}} \cdot \Delta T}. \tag{17}$$

We note that in the biphasic kinematics contribution model, we only take the vaporization into consideration and ignore the condensation process, because the frequency of bubble-rising generated by vaporization is much higher than that of the droplet-falling generated by condensation.

Supplementary Note 7: Evidence of heat transfer enhancement by active particles induced liquid agitation

The passive scalar transport can be modeled as an effective diffusive process [3]. By definition, Nusselt number is the ratio of effective heat transport coefficient to the pure thermal conduction

coefficient, which means

$$\begin{aligned}
\mathrm{Nu} &= \frac{\lambda_{\mathrm{e}}}{\lambda} \\
&= \frac{\kappa_{\mathrm{e}} \cdot \rho \cdot C_{\mathrm{p}}}{\kappa \cdot \rho \cdot C_{\mathrm{p}}} \\
&= \frac{\kappa_{\mathrm{e}}}{\kappa}.
\end{aligned} \tag{18}$$

where λ_{e} is the effective heat transport coefficient, λ pure thermal conduction coefficient, κ_{e} the effective diffusivity, κ thermal diffusivity of the working fluid, ρ the density of the working fluid, C_{p} the heat capacity per unit mass of working fluid.

The Galileo number Ga was measured to be larger than 1000. In this regime, Ga is comparable to the particle Reynolds number Re_{p} [4, 5]. This made us estimate that the particle wakes are turbulent [5–8].

At low vapor phase of biphasic species volume fraction ($< 3\%$), the effective diffusivity κ_{e} can be modeled as $\kappa_{\mathrm{e}} \propto u'\Lambda$, where u' is theoretically estimated liquid velocity fluctuation (r.m.s), Λ the integral length scale of the liquid velocity fluctuations. Λ is a function of bubble diameter d and rising velocity v of a single bubble, so when α changes, Λ does not change. If the turbulence is a result of active particle induced agitation, then the induced agitation by the biphasic particles can be modeled as $u' \approx V_{\mathrm{c}}\alpha^{0.5}$, where V_{c} is the collective active particle velocity, so the Nusselt number should have a linear dependence on $V_{\mathrm{c}}\alpha^{0.5}$ [3, 9, 10]. After plotting the differential heat flux enhancement $\delta\mathrm{Nu} - \mathrm{Nu}_{\mathrm{l}}$ against $V_{\mathrm{c}}\alpha^{0.5}$, we observe the linear growth which provides further evidence that the induced agitation (in water) by the biphasic particles accounts for the remainder of the heat exchange , which is the major enhancement. Thus, the effective heat transfer gain of biphasic turbulence is believed to result from the combined contributions of (a) the kinematics of the active particles, and (b) their induced liquid agitation

Supplementary Note 8: Linear scalings of heat exchange with temperature change from different experimental settings

Through the constant ΔT experiment, the system shows regimes of linear scalings of heat exchange with temperature change. We want to further see whether there will also be regimes of linear scalings of heat exchange with temperature change when we make independent changes

in bottom (case 2) and top (case 3) plate temperatures or change the geometry property (case 4) of the experimental cell (i.e. aspect ratio) , so we make other two heat transfer measurements (case 2 is at the condition of constant bottom plate temperature (also $\mathrm{Ja_b}$) and changing the top plate temperature (also $\mathrm{Ja_t}$); case 3 is at the condition of constant top plate temperature (also $\mathrm{Ja_t}$) and changing the bottom plate temperature (also $\mathrm{Ja_b}$)) and case 4 is visualization for experimental setup of aspect ratio 1 (see Suppl. Video 11).

Here, we show case 2 and case 3 in Supplementary Figure. 11. Supplementary Figure. 11 (a)-(c) are for case 2 (the bottom plate temperature is $T_\mathrm{b} = 46.7°\mathrm{C}$ and fixed, we only change the top plate temperature T_t) and Supplementary Figure. 11 (d)-(f) are for case 3 (the top plate temperature is $T_\mathrm{t} = 13.2°\mathrm{C}$ and fixed, we only change the bottom plate temperature T_b). As shown in Supplementary Figure. 11, with independent change of top or bottom plate temperature, we can also observe the regimes of linear scalings of heat exchange with temperature change.

For case 2, as T_t increasing, the driving for condensation is decreasing, while the bulk region temperature is increasing, we observe Nu is increasing with increasing $\mathrm{Ja_t}$ and reaches the highest Nu which is more than 500% times that of the quiescent regime. During the experiment, the vapor phase of biphasic species volume fraction remains almost unchanged and is comparable to the highest α in case 1, which means nearly all the HFE-7000 takes part in the convection system.

For case 3, as T_b increasing, the driving for vaporization is increasing, and the bulk region temperature is also increasing, we observe an increment of Nu with $\mathrm{Ja_b}$, and the highest heat flux enhancement is 300% more than of the quiescent regime. α is at first increases and then reaches a plateau, which means as T_b increasing, the system changes from partially active to fully active.

For case 4, we use the experimental setup with aspect ratio 1 (d = H = 200 mm). Here we just show qualitative experiment observations of such experimental setting (see Suppl. Video 11) to prove that the biphasic activity concept is also efficient in other geometry cell.

Cases 2-4 further justify that the system shows regimes of linear scalings of heat exchange with temperature change (for independent changes in bottom and top plate temperatures), and for aspect ratio changes, suggesting robust predictability and heat transfer control in both heating and cooling applications.

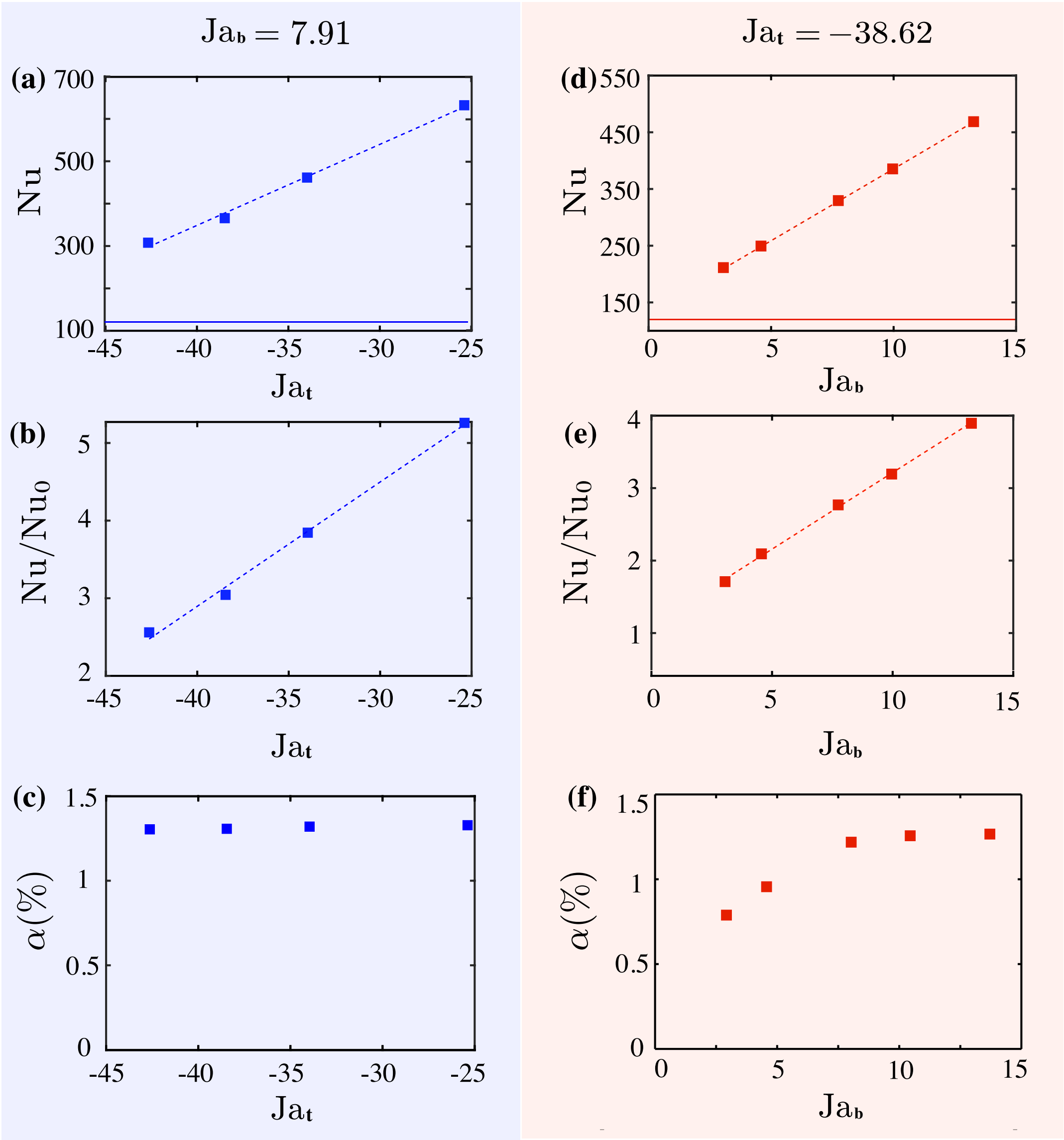

Supplementary Figure 11. The experimental results for for case 2 (fixed superheat) and case 3 (fixed underheat). (a)The Nusselt number Nu for case 2 as a function of $\mathrm{Ja_t}$. The dashed line is the guide to the eye. (b) Nusselt number Nu compensated by one-phase Nusselt number $\mathrm{Nu_0}$ fro case 2. The dashed line is the guide to the eye. (c) Vapor phase of biphasic species volume fraction α for case 2 as function of $\mathrm{Ja_t}$. (d)The Nusselt number Nu for case 3 as a function of $\mathrm{Ja_t}$. The dashed line is the guide to the eye. (e) Nusselt number Nu compensated by one-phase Nusselt number $\mathrm{Nu_0}$ for case 3. The dashed line is the guide to the eye. (f) Vapor phase of biphasic species volume fraction α for case 3 as function of $\mathrm{Ja_t}$.

Supplementary Note 9: Image processing

Images of biphasic bouncing and biphasic migration are generated by superimposing several particle trajectories through MATLAB processing. First we read the videos into Matlab and adjust the contrast of every frame to get a good view. Then we estimate the bouncing (migration) time scale (during such time period several trajectories of total life time can be observed). For

the same one particle, within the characteristic time scale, can be observed in each frame. In order to freeze the position for the same one particle along its trajectory we superimpose all the frames and then get the average image of the summation. The average image of the summation is what can be seen from Supplementary Figure. 3(d) and (f) in the paper.

The collective velocity is analyzed by using the concept of PIV (Particle Image Velocimetry) code [11, 12]. The camera was used to take videos on the full flow field in the cell. The biphasic particles in the active regime is similar to the function of the tracer particle in PIV experiment. The videos are read in MATLAB in the form of frames and then all the frames are split into small observed windows (32 pixels$\times$ 32 pixels). We scan the window one by one and calculate the cross correlation between the adjacent frame in the time sequence, and therefore we can get the displacement vector for the image pair. The displacement can be converted into velocity by dividing the time interval of the displacement. For the heat transfer in the current problem, we only care about the velocity in the vertical direction, so we only calculate the vertical velocity along the horizontal cross section of the cell. Then we estimate the scale of the vertical velocity which is used to calculate the biphasic kinematic contribution to the heat transfer enhancement.

Supplementary References

[1] M. H. Rausch, L. Kretschmer, S. Will, A. Leipertz, and A. P. Froba, *Density, surface tension, and kinematic viscosity of hydrofluoroethers HFE*−7000*, HFE*−7100*, HFE*−7200*, HFE*−7300*, and HFE*−7500, J. Chem. Eng. Data **60**, 3759 (2015).

[2] M. Snare, F. Treloar, K. Ghiggino, and P. Thistlethwaite, *The photophysics of rhodamine B*, J. Photochem. **18**, 335 (1982).

[3] E. Alméras, F. Risso, V. Roig, S. Cazin, C. Plais, and F. Augier, *Mixing by bubble-induced turbulence*, J. Fluid Mech. **776**, 458 (2015).

[4] P. Ern, F. Risso, D. Fabre, and J. Magnaudet, *Wake-induced oscillatory paths of bodies freely rising or falling in fluids*, Annual Review of Fluid Mechanics **44**, 97 (2012).

[5] M. Horowitz and C. H. Williamson, *Vortex-induced vibration of a rising and falling cylinder*, Journal of Fluid Mechanics **662**, 352 (2010).

[6] M. Horowitz and C. Williamson, *Critical mass and a new periodic four-ring vortex wake mode for freely rising and falling spheres*, Physics of Fluids **20**, 101701 (2008).

[7] V. Mathai, X. Zhu, C. Sun, and D. Lohse, *Flutter to tumble transition of buoyant spheres triggered by rotational inertia changes*, Nature communications **9**, 1792 (2018).

[8] E. Almras, V. Mathai, D. Lohse, and C. Sun, *Experimental investigation of the turbulence induced by a bubble swarm rising within incident turbulence*, Journal of Fluid Mechanics **825**, 1091 (2017).

[9] G. Riboux, F. Risso, and D. Legendre, *Experimental characterization of the agitation generated by bubbles rising at high Reynolds number*, J. Fluid Mech. **643**, 509 (2010).

[10] B. Gvozdić, E. Alméras, V. Mathai, X. Zhu, D. P. van Gils, R. Verzicco, S. G. Huisman, C. Sun, and D. Lohse, *Experimental investigation of heat transport in homogeneous bubbly flow*, J. Fluid Mech. **845**, 226 (2018).

[11] R. J. Adrian, *Particle-imaging techniques for experimental fluid mechanics*, Annu. Rev. Fluid Mech. **23**, 261 (1991).

[12] J. Westerweel, G. E. Elsinga, and R. J. Adrian, *Particle image velocimetry for complex and turbulent flows*, Annu. Rev. Fluid Mech. **45**, 409 (2013).
