# Supplementary material for: Self-sustained biphasic catalytic particle turbulence
Source: Nat Commun. 2019 Jul 26;10:3333. doi: 10.1038/s41467-019-11221-w (PMC6659658; doi:10.1038/s41467-019-11221-w)
Supplement: Supplementary file 3 — Description of Additional Supplementary Files [file 41467_2019_11221_MOESM3_ESM.pdf]

Description of Additional Supplementary files

Supplementary Movie 1 : Biphasic risers and settlers.

The movie shows the novel heat carriers in the bulk region of the active biphasic particle turbulence system. The movie plays at 0.2 $\times$.

Supplementary Movie 2 : Biphasic plumes.

The movie shows the novel heat carriers near the bottom plate of the active biphasic particle turbulence system. The movie plays at 0.2$\times$.

Supplementary Movie 3 : Classical thermal turbulence system.

The movie shows shadowgraphy images of the classical thermal turbulence system in which we can see heat transporters (hot and cold thermal plumes). The movie plays at 16 $\times$.

Supplementary Movie 4 : Partially active regime of the whole flow field.

Partially active regime (0 K $< T_{\mathrm{b}} - T_{\mathrm{cr}} <$5 K). In this regime there is still a fraction of HFE-7000 is in liquid phase that spreads on the bottom plate, and a small fraction of HFE-7000 takes part in the biphasic activity. The movie plays at real time.

Supplementary Movie 5 : Fully active regime of the whole flow field.

Fully active regime ($T_{\mathrm{b}} - T_{\mathrm{cr}} >$ 5 K). All HFE-7000 takes part in the activity, but this does not mean all

HFE-7000 liquid has become vapor, instead all HFE-7000 has joined the bulk region and contributes to the heat transfer enhancement (in fact there s only small fraction of HFE-7000 liquid becomes vapor). In this movie, $T_b \approx 51$ K, $T_t \approx 21$ K, we can observe the "sweeping mode". The movie plays at 0.5×.

Supplementary Movie 6 : Zoom-in bottom plate (bubble plumes) of partially active regime.

This movie is a zoom-in view of the bottom plate with the same setting of Supplementary Movie 4. The movie plays at real time.

Supplementary Movie 7 : Zoom-in bottom plate (sweeping mode) of fully active regime.

This movie is a zoom-in view of the bottom plate with the same setting of Supplementary Movie 5. We can clearly observe the "sweeping mode". The movie plays at real time.

Supplementary Movie 8 : Biphasic bouncing.

The biphasic active fluid motion is limited to the lower half of the convection setup. The movie plays at real time.

Supplementary Movie 9 : Biphasic migration.

Biphasic migration regime is observed at a higher activity level. Here the particles migrate from bottom plate to top plate and vice versa. The movie plays at real time.

Supplementary Movie 10 : Mixing by turbulent plumes.

The movie shows the mixing of a patch of fluorescent dye (passive scalar) released in classical thermal turbulence system (false coloring was adopted based on the intensity of the dye).

Supplementary Movie 11 : Mixing by active biphasic turbulence.

The movie shows the mixing of a patch of fluorescent dye (passive scalar) released in active biphasic turbulence (false coloring was adopted based on the intensity of the dye). Supplementary Movies 10 & 11 are accelerated by 15 times compared to the real time.

Supplementary Movie 12 : Active biphasic particle turbulence system of aspect ratio 1.

All the experimental conditions in the system of aspect ratio 1 are the same as the aspect ratio 0.5 experiments except for the aspect ratio, in this movie the aspect ratio equals 1 which means the thickness/height of the working fluid layer (water) and the diameter of the cell are the same. The movie plays at 4.0 ×.
